# Impact of Obstetric Complications in Subjects at Clinical High Risk for Psychosis: A Systematic Review and Meta‐Analysis

**DOI:** 10.1111/acps.13816

**Published:** 2025-05-04

**Authors:** Inmaculada Baeza, Jordina Tor, Elena de la Serna, Gisela Sugranyes, Fàtima Crispi, Montserrat Izquierdo‐Renau, Marta del Olmo, Montserrat Dolz, Clemente García‐Rizo

**Affiliations:** ^1^ Department of Child and Adolescent Psychiatry and Psychology, SGR2021‐01319, Institute of Neuroscience Hospital Clínic of Barcelona Barcelona Spain; ^2^ Agustí Pi i Sunyer Biomedical Research Institute (CERCA‐IDIBAPS) Barcelona Spain; ^3^ Mental Health Networking Biomedical Research Centre (CIBERSAM), ISCIII Madrid Spain; ^4^ Institute of Neurosciences University of Barcelona Barcelona Spain; ^5^ Sant Joan de Déu Research Institute Barcelona Spain; ^6^ Department of Child and Adolescent Psychiatry and Psychology Sant Joan de Déu Hospital Barcelona Spain; ^7^ BCNatal (Hospital Clínic of Barcelona and Sant Joan de Déu Hospital), Maternal‐Fetal Medicine Barcelona Spain; ^8^ Rare Disease Networking Biomedical Research Centre (CIBERER), ISCIII Madrid Spain; ^9^ Neonatology Department Sant Joan de Déu Hospital Barcelona Spain; ^10^ Barcelona Clinic Schizophrenia Unit, Institute of Neuroscience Hospital Clínic de Barcelona Barcelona Spain

**Keywords:** clinical high risk, obstetric complications, outcome, pregnancy and delivery, psychosis risk, psychotic disorder, transition to psychosis

## Abstract

**Introduction:**

Exposure to obstetric complications (OCs) increases the risk of developing psychosis and schizophrenia in offspring. However, studies with subjects at clinical high risk for psychosis (CHR) have reported inconsistent results. We conducted a systematic review and meta‐analysis to evaluate the prevalence of OCs among CHR subjects and controls and examine their impact on the transition to psychosis.

**Methods:**

Four databases (Web of Science, PubMed, Latindex, and Dialnet) were systematically searched for articles published between 1995 and June 6, 2024. The risk of bias was assessed using the Newcastle–Ottawa scale. Articles providing data on OCs in CHR subjects were included.

**Results:**

A total of 6037 records were retrieved through systematic and citation searches. Nine articles met the inclusion criteria for our systematic review and provided data for meta‐analysis. A total of 555 CHR participants were included. Meta‐analysis showed a significantly higher prevalence of OCs in CHR subjects versus controls: RR = 1.45 (95% CI: 1.16, 1.81), (*Z* = 3.27, *p* = 0.0011). Data from three longitudinal studies assessed transition to psychosis and our meta‐analysis found a trend toward an increased risk of transition in CHR subjects with a history of OCs compared to others: RR = 2.05 (95% CI: 0.98, 4.26), *Z* = 1.91, *p* = 0.056.

**Conclusions:**

CHR for psychosis was associated with OCs, though their role in the transition to psychosis requires further study. OCs should be recorded and analyzed in CHR individuals, considering their potential clinical implications.

**Summary**

- Summation
  ○ OCs were more prevalent in CHR for psychosis subjects compared to controls.
  ○ Abnormal gestational age and cesarean delivery were the most prevalent definite or equivocal OCs found in CHR for psychosis subjects.
  ○ The reviewed studies were of high quality according to the Newcastle–Ottawa scale.
- Limitations
  ○ The small sample sizes of some of the studies included in the review.
  ○ Only three studies were longitudinal and results regarding the impact of OCs in transition to a psychotic disorder were not conclusive.
  ○ Most of the studies used the Lewis–Murray scale to measure OCS, and each of its subscales includes a heterogeneous set of obstetric events.

## 1 Introduction

Because the personal and societal costs of psychotic disorders are high [1, 2], increased efforts have been made in recent years to find ways to prevent these conditions from developing [3]. The concept of clinical high risk for psychosis (CHR) was coined almost 30 years ago [4] to refer to subjects with a high risk of developing a first episode of a psychotic disorder. This classification has become widespread, being consistently recommended [5] and used in early detection services [6], based on clinical criteria including genetic risk and/or attenuated manifestations of symptoms. In a meta‐analysis, the transition to psychosis from a CHR state was observed to be 15% at 1 year, and 25% after 3 years [7]. However, to date, only attenuated positive psychotic symptoms, global functioning, and negative psychotic symptoms have shown suggestive evidence in a meta‐analysis for an association with the transition to psychosis [8]. This signals a need to evaluate other potential risk factors which could help improve early detection and the prompt implementation of appropriate intervention strategies.

It is well‐known that the underlying cause of developing a psychotic disorder includes the result of an interaction between genetic load and environmental factors [9, 10] according to both the neurodevelopmental hypothesis [11, 12, 13] and the developmental risk factor model [14]. Among the environmental factors, the presence of obstetric complications (OCs) has been historically described to play a key role. Although all OCs are usually included as a single group, they can be quite different in terms of timing and outcome, as they range from difficulties during pregnancy and abnormal fetal growth and development to complications during delivery [15, 16]. OCs increase the risk of developing a psychotic disorder between 1.4 and 3.05 times compared to controls [17, 18] and have been included in an environmental risk score for psychosis [19]. OCs have been associated with different outcomes related to psychosis as epiphenomena: earlier onset of the first episode of psychosis (FEP) [20, 21], more severe symptomatology in patients with schizophrenia [22], cognitive effects such as abnormalities in verbal and working memory [23], metabolic effects such as increased weight‐gain in low birth weight patients [24] and brain structural abnormalities [25].

Some OCs are associated with an increased risk of developing nonclinical psychosis‐like symptoms in adolescents of the general population [26]. Some studies have also linked OCs to an increased risk of developing a CHR state [27, 28], though these findings are inconsistent [29, 30]. Nevertheless, unspecified OCs have been reported to increase the risk of CHR threefold [31]. In contrast, the role of OCs in the transition to a full‐blown psychotic disorder (CHR‐T) is not clearly understood [30, 32].

There are different ways in which the consequences of OCs in the offspring could lead to psychosis later in life. For instance, in pregnancy‐related complications (bleeding, severe preeclampsia, syphilis or rubella, and rhesus incompatibility), an increased inflammation and oxidative stress state has been described in preeclampsia [33] and bleeding [34]. Looking at abnormal fetal growth and development difficulties (low birth weight, twin delivery, abnormal gestational age pre‐ or post‐term, and congenital malformations), most of these are associated with hypoxia, while inflammation is also one of the underlying reasons in most of these cases [35, 36]. In contrast, intrauterine growth restriction in preterm subjects is associated with alterations of brain metabolites and structure [37].

Regarding complications of delivery (premature rupture of membranes, long duration of delivery, umbilical cord prolapse, complicated cesarean delivery, abnormal fetal presentation, and use of forceps), both hypoxia and inflammation are also associated with these OCs [34]. Indeed, birth asphyxia was found to be directly associated with brain structural changes and cognitive impairment in psychosis patients [38].

Prenatal hypoxia alters dopamine neurons' development, as animal models have demonstrated [39, 40], and other neurotransmitters could also be dysregulated [41]. The dopaminergic system is considered key in the progression toward psychosis, while other possible mechanisms, such as inflammation, could also affect dopamine regulation [42]. Moreover, inflammation could affect glutamate, which may play a role in the development of psychosis [43].

The relationship between developmental stages during uterine life and posterior outcomes in adolescence or adulthood are included in the “Fetal origins of adult disease” theory, first explained by Barker [44] and later embedded in the developmental origins of health and disease model (DOHaD) [45]. According to DOHaD, intrauterine signals program the development of tissue and can create specific risks for chronic diseases and could help explain the relationship found between OCs and both CHR and CHR‐T. Thus, a systematic review evaluating the prevalence of OCs in CHR, and their impact on transition to psychosis could help clarify current knowledge.

The main objective of the present systematic review and meta‐analysis is to summarize findings regarding the prevalence of OCs among CHR versus controls and their impact on the clinical outcomes of CHR subjects.

## 2 Materials and Methods

### 2.1 Literature Search

To perform this systematic review, we followed the 2021 Preferred Reporting Items for Systematic review and Meta‐Analyses (PRISMA) guidelines [46]. Its protocol was registered in the International Prospective Register of Systematic Reviews (PROSPERO) with the number: CRD42024553492.

A systematic literature search was carried out in Web of Science, PubMed, Latindex, and Dialnet databases from 1995 to June 6, 2024, using the following terms: (OCs or labor or birth or delivery or pregnancy) and (psychosis risk or CHR or ultra high risk for psychosis or at‐risk mental state for psychosis).

### 2.2 Inclusion Criteria

The criteria for selecting the articles were the following: (1) original articles; (2) providing data on OCs in CHR subjects and comparison to controls or in the CHR subjects' transition and outcomes; (3) written in English or Spanish.

Although not meeting our original inclusion criteria, three additional articles were found and included as these provided data comparing CHR subjects to FEP patients. Data were analyzed and included in the results as a secondary outcome.

### 2.3 Exclusion Criteria

Reviews and meta‐analyses, animal studies, case reports or series, clinical guides, expert consensus, and non‐peer‐reviewed literature such as book chapters, PhD theses, posters, or oral communication in conferences. Grey literature was not searched.

### 2.4 Data Systematization

The search was conducted in three steps: first, two researchers (I.B. and J.T.) performed the electronic search. Secondly, both reviewed one by one all of the references from the selected articles using the web platform www.rayyan.ai to help with duplicates and classification. When there was a discrepancy, the article was discussed together with a third author (CG‐R). In the last step, I.B. and J.T. extracted the following data from the studies: bibliographic references, the place where the study was conducted, year of publication, study design, description of the sample (number, sex, age, criteria for CHR, criteria for controls), OC scales or measures, the prevalence of OCs, diagnosis and outcome at follow‐up, as well as predictors of outcome.

The authors of studies were contacted in cases where information about the classification or prevalence of OCs was missing in the article.

### 2.5 Quality of Assessment

The quality of assessment of each study was evaluated with the Newcastle–Ottawa scale for case–control or cohort studies [47] with study‐specific criteria [48] (Tables S1 and S2).

### 2.6 Data Analysis

We conducted a meta‐analysis when the studies reviewed met the following criteria: three or more studies measured OCs with a healthy comparison group or another comparison group, and the results regarding the percentage of OCs and the number of subjects in the sample were published. As a result, meta‐analyses for studies on the prevalence of OCS in CHR versus healthy control (HC) participants, CHR versus FEP participants, and CHR‐T versus CHR who did not transit to a psychotic disorder (CHR‐NT) were performed. The estimated effects for the outcomes were reported with 95% confidence intervals (CIs) and computed with random effects and common effects models. Heterogeneity was estimated according to the *I* ^2^ statistic; values < 25% were considered to represent low heterogeneity, 25%–50% moderate heterogeneity, and > 75% high heterogeneity. Egger's test was used to assess the funnel plot asymmetry. All tests were two‐tailed, and *p* < 0.05 was considered significant. Meta‐analysis was performed using the “meta” package version 7.0‐0. All analyses were performed using R Statistical Software (v 4.4.1; R Core Team 2024, Vienna, Austria).

## 3 Results

Out of 6037 articles retrieved, nine were included, eight by meeting eligibility criteria, while one was found through snowballing (from the references of another article). All of the included articles were reviewed in depth and, taken together, examine a total of 555 CHR participants.

**FIGURE 1:**
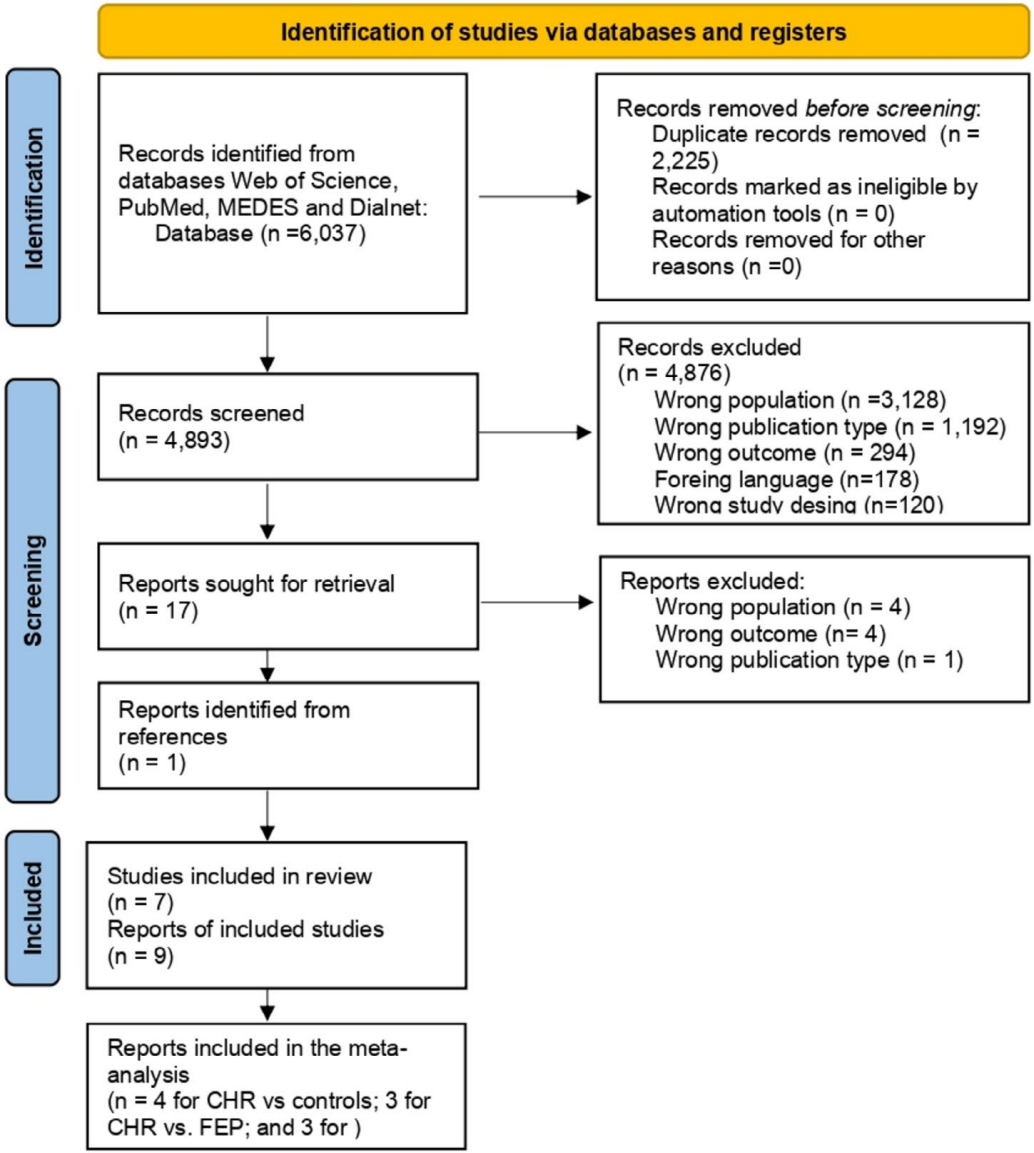
PRISMA 2020 flow diagram for new systematic reviews that include searches of databases and registers only. CHR, clinical high risk; FEP, first episode of psychosis. *Source*: Page et al. [46].

The flowchart of the process and the reasons for exclusion are shown in Figure 1.

The included articles examine the results of seven different studies; because two were from the same cohort but with different sample sizes, the first being a baseline comparison and the second showing the follow‐up results [28, 29, 30, 49]. Two studies (33.3%) were conducted in the United States [27, 50], and one in each of the following countries: Australia [32], Italy [51], Poland [28, 49], and Spain [29, 30]. There was also a multicenter study that was conducted in Finland, Germany, the Netherlands, and the United Kingdom [52].

Of the nine articles included, three (33.3%) had cross‐sectional designs [27, 28, 29] and the others were longitudinal [30, 32, 49, 50, 51]. Four of the longitudinal studies included only CHR samples without a control or comparison group [32, 49, 51]. Table 1 summarizes the principal characteristics of the reviewed studies.

**TABLE 1:** Main characteristics of the included studies (*N* = 9), in chronological order of publication.

	Study/country/setting	Year	Type of study	*N* and diagnosis	CHR criteria	Mean age (years)	OCS assessment	Results
1	Yun et al./Australia/University of Melbourne [32]	2005	Longitudinal	74 CHR	PACE. One of the following: (1) attenuated psychotic symptoms during the past year; (2) brief limited intermittent psychotic symptoms: have experienced episodes of frank psychotic symptoms that did not last longer than 1 week and spontaneously abated; or (3) trait and state risk factor: having a first degree relative with a psychotic disorder or the identified patient has a schizotypal personality disorder and experienced a significant decrease in functioning during the previous year	At baseline, 18.85 ± 3.62	Lewis–Murray obstetric complication scale Season of birth (winter or non‐winter); place of birth (rural or urban); maternal age; number of previous pregnancies; parity; illnesses during the pregnancy; and medication use during pregnancy	OCS were not associated with the later development of a psychotic disorder after 1 year follow‐up
2	Ballon et al./USA/University of California San Diego [27]	2008	Cross‐sectional	52 CHR 18 FES 43 Controls	Modified SIPS criteria: one of the following: (1) attenuated positive symptom syndrome; (2) brief limited intermittent psychotic symptom syndrome; genetic risk (first or second degree relative with a history of any psychotic disorder) or schizotypal personality disorder in subject and deterioration syndrome	CHR 18.4 FES 20.2 HC 20.2	Lewis–Murray obstetric complication scale Maternal and paternal age at birth; and maternal substance use during pregnancy	Definite or equivocal OCS were increased in CHR (46%) and FES (39%) samples compared to the control (19%) group, but statistical significance was only found between CHR and controls
3	Mittal et al./USA/University of California Los Angeles [37]	2009	Longitudinal prospective	47 CHR	SIPS. One of the following: (1) attenuated positive symptom syndrome; (2) brief limited intermittent psychotic symptom syndrome; genetic risk (first degree relative with a history of any psychotic disorder) or schizotypal personality disorder in subject and deterioration syndrome	16.32 ± 2.88	Lewis–Murray obstetric complication scale	A history of OCs was associated with increased odds of conversion to psychosis (OR = 4.90, CI: 1.04/22.20). There was a moderate positive relationship between OCS and total prodromal symptoms at baseline
4	Preti et al./Italy/Azienda Ospedaliera Ospedale Niguarda Ca' Granda, Milano [38]	2012	Longitudinal retrospective	82 CHR 86 FEP	PACE	At baseline, CHR‐P 22.3 ± 3.6 FEP 22.5 ± 3.8	Interview with mothers with an ad hoc list of OCS	No differences were found in the prevalence of OCS between CHR and FEP nor among CHR‐P and CHR‐NP, after a 1 year follow‐up. In the CHR group, patients younger than 21 were more likely to have a history of OCS than older patients
5	Korkeila et al./Finland/University of Turku [39]	2013	Longitudinal prospective	245 CHR	CROP. One of the following: (1) attenuated psychotic symptoms, (2) brief limited intermittent psychotic symptoms, (3) genetic risk and decreased functional level, or (4) at least two moderate symptoms in the Bonn Scale for the Assessment of Basic Symptoms Prediction List (BSABS‐P, 8) were present	At baseline, 22.6 ± 5.1	Basic data form created for the EPOS study, which included information about obstetric complications	39/239 (16.3%) participants presented OCs. Those with OCS had significantly more physical symptoms or an illness than those without them. No data were reported about OCs in the 33 patients who transited to a psychotic disorder
6	Kotlicka‐Antczak et al./Poland/Medical University of Lodz [28]	2014	Cross‐sectional	66 CHR 50 FES 50 HC	PACE using the CAARMS	CHR‐P 18.58 ± 3.31 FES 19.84 ± 2.74 HC 19.06 ± 2.28	Lewis–Murray obstetric complication scale Apgar score 1 and 5 min after birth	Definite OCS occurred significantly more frequently in the CHR group compared to HC (OR = 4.20, 95% CI = 1.46–12.11), as well as in the FES subjects compared to HC (OR = 4.64, 95% CI = 1.56–13.20). CHR subjects had significantly lower Apgar 5 scores compared to HC
7	Kotlicka‐Antczak et al./Poland/Medical University of Lodz [36]	2018	Longitudinal prospective	82 CHR	PACE using the CAARMS	At baseline, CHR‐P 18.6 ± 3.4	Lewis–Murray obstetric complication scale Apgar score 1 and 5 min after birth	A history of at least one definite obstetric complication was associated with increased risk of transition to schizophrenia (OR = 6.57, 95% CI = 1.89–22.85) after a mean follow‐up of 42.3 ± 28.3 months
8	Dolz et al./Spain/Hospital Clinic and Hospital Sant Joan de Déu [29]	2018	Cross‐sectional	91 CHR 45 HC	SIPS including other criteria: (1) attenuated positive symptoms; (2) attenuated negative symptoms in the previous 12 months; (3) brief intermittent limited psychotic symptoms; and (4) genetic risk (first or second degree relative with schizophrenia) or schizotypal disorder and impairment of functioning	CHR‐P 15.5 ± 1.5 HC 15.1 ± 1.7	Lewis–Murray obstetric complication scale	No differences were found in any OCS between CHR and HC, but CHR‐P subjects scored higher on gestational age (42 weeks) than HC. The most frequent OCS in CHR‐P subjects was abnormal gestational age, forceps delivery, and cesarean section
9	Dolz et al./Spain/Hospital Clinic and Hospital Sant Joan de Déu [30]	2024	Longitudinal prospective	101 CHR 110 HC	SIPS including other criteria: (1) attenuated positive symptoms; (2) attenuated negative symptoms in the previous 12 months; (3) brief intermittent limited psychotic symptoms; and (4) genetic risk (first or second degree relative with schizophrenia) or schizotypal disorder and impairment of functioning	At baseline, CHR‐P 15.2 ± 1.8 CHR‐NP 15.3 ± 1.6 HC 15.7 ± 1.6	Lewis–Murray obstetric complication scale	No differences were found in definite or definite + equivocal OCS between CHR and HC or between CHR‐P and CHR‐NP. OCS were not associated with developing a psychotic disorder after 1.5 years follow‐up

Abbreviations: CAARMS, Comprehensive Assessment of At‐Risk Mental State; CHR, clinical high risk for psychosis; CHR‐NP, clinical high risk who do not transit to a psychotic disorder; CHR‐P, clinical high risk who transited to a psychotic disorder; CROP, current risk of psychosis; EPOS, European Prediction of Psychosis Study; FEP, first episode of psychosis; FES, first episode of schizophrenia; HC, healthy controls; OCS, obstetric complications; PACE, personal assessment and crisis evaluation; SIPS, structured interview for prodromal syndromes; USA, United States of America.

### 3.1 Clinical High Risk for Psychosis Criteria

Seven studies used different criteria for determining CHR for psychosis status: The Personal Assessment and Crisis Evaluation (PACE) criteria was used by four studies [28, 32, 49, 51], with two also using the Comprehensive Assessment of At‐Risk Mental State (CAARMS) tool [28, 49]. The structured interview for prodromal syndromes (SIPS) criteria was adopted by the other three studies [27, 29, 30, 50, 52]. One of these [29, 30] used the SIPS criteria plus attenuated negative symptoms and a genetic risk criterion with a first or second degree relative with schizophrenia. Another study [27] utilized a modified version of the SIPS which also included a genetic risk criterion with a first or second degree relative with schizophrenia. The third study [52] combined the SIPS criteria together with two basic symptoms and referred to this as current risk of psychosis (CROP) criteria. All are described in Table 1.

### 3.2 Obstetric Complications Measures

Seven (87.5%) articles [27, 28, 29, 30, 32, 49, 50] used the Lewis–Murray obstetric complication scale [53] to measure OCs, with four of them complementing this with other information related to pregnancy and birth [27, 28, 32, 49]. The Lewis–Murray obstetric complication scale consists of 15 complications, divided into prenatal (four items) and peri‐postnatal complications (11 items), which are rated as definite or equivocal [41]. The Lewis–Murray scale can be grouped into three categories [15, 16]: Lewis A—complications of pregnancy (syphilis or rubella, rhesus isoimmunization/Rh incompatibility, severe preeclampsia, requiring hospitalization or induction of labor, and bleeding before delivery or threatened abortion); Lewis B—abnormal fetal growth and development (twin delivery, abnormal gestational age including preterm birth before 37 weeks or long‐term after 42 weeks, weight at birth less than 2500 g, and any important physical abnormality); and Lewis C—difficulties in delivery (premature rupture of membranes, duration of delivery more than 36 h or less than 3 h, umbilical cord prolapse, complicated cesarean delivery, abnormal fetal presentation, use of forceps, and being in an incubator for more than 4 weeks).

Most of the articles described definite and equivocal OCs separately [28, 30, 49], although some grouped these together [27, 29], and one included only definite complications [50]. One study included seven additional OC items that are not included in the Lewis–Murray scale [32]. Another article was based on an ad hoc list of OCs [51], while one [52] used the basic data form created for the European Prediction of Psychosis Study (EPOS) [54].

### 3.3 Types of OCs in CHR

The most common OCs found in CHR subjects were abnormal gestational age (7.7%–29.2%) and cesarean delivery (4.8%–24.2%). When taking into account only definite OCs, abnormal gestational age (2.1%–11.9%) and cesarean delivery (6.6%–17%) were the most prevalent.

Tables S3 and S4 show percentages of OCs (any and definitive) among all studies.

### 3.4 OCs Prevalence Among CHR and Healthy Control Participants

Four studies described data on the comparison of rates of OCs between CHR and HC participants [27, 28, 29, 30] (Table 1). Percentages of subjects presenting any OCs (definite or equivocal) vary between 46.2% and 55.4% in CHR and between 18.6% and 48% in HC.

**FIGURE 2:**
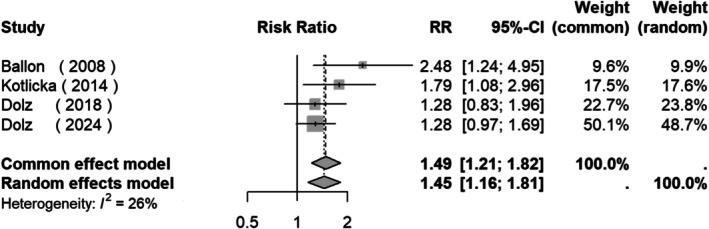
Forest plot of studies measuring the prevalence of obstetric complications between clinical high risk for psychosis subjects and controls.

A meta‐analysis was performed with these studies and the results indicate that there was a significant overall difference in the prevalence of OCs between patients and HC: RR = 1.45 (95% CI: 1.16, 1.81, *Z* = 3.27, *p* = 0.0011; Figure 2). Funnel plots were drawn for the meta‐analysis, showing no asymmetry. Egger's tests were performed and, again, no significant asymmetry was found (Figure S1).

### 3.5 Differences in OCs Among CHR According to Transition to Psychosis (CHR‐T vs. CHR‐NT)

Five studies with longitudinal design compared OCs between CHR‐T and CHR‐NT [30, 32, 49, 50, 51]. Among those reporting data of general OCs prevalence, at least one OC was found in 59%–89% of the CHR‐T sample, and only 48.4%–64% of the CHR‐NT sample [30, 49].

One study which specifically analyzed each OC found that lower birth weight was higher in CHR‐T versus CHR‐NT (3.49 ± 0.60 vs. 2.89 ± 0.79 kg) [32]. Nevertheless, in the same study, neither intrauterine growth restriction complications (including low birth weight and preterm delivery) nor acute hypoxia complications (breech or other abnormal presentation, long duration of labor, caesarian delivery, and need for neonatal incubator) were associated with transition to psychosis [32].

**FIGURE 3:**
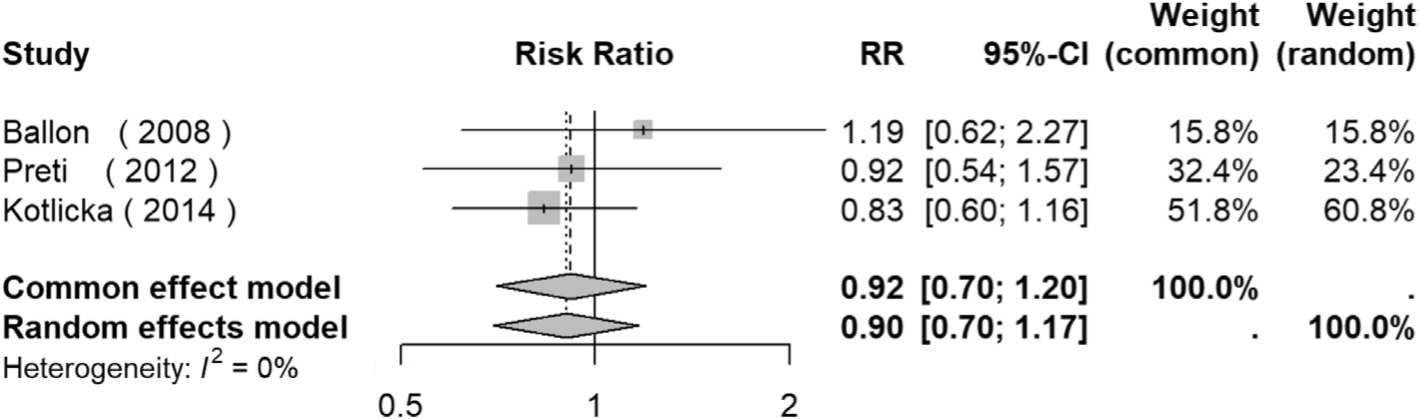
Forest plot of studies measuring the prevalence of obstetric complications between clinical high risk for psychosis subjects at baseline and clinical high risk for psychosis subjects who transitioned to a psychotic disorder.

The meta‐analysis of the three studies with available data of total OCs [30, 49, 50] showed a tendency toward a positive association between OCs and CHR‐T: RR = 2.05 (95% CI: 0.98, 4.26, *Z* = 1.91, *p* = 0.056; Figure 3).

### 3.6 Secondary Outcome: OCs Prevalence Between CHR and FEP Participants

Three studies compared OCS prevalence between CHR and FEP participants [27, 28, 51]. Percentages of any OCs in the FEP group varied between 23% and 60%. Two of the studies analyzed a sample of participants with a diagnosis of First episode of Schizophrenia in the last year [27, 28], and other participants with a first episode of schizophrenia or related syndromes (F20–29 in ICD‐10) [51].

**FIGURE 4:**
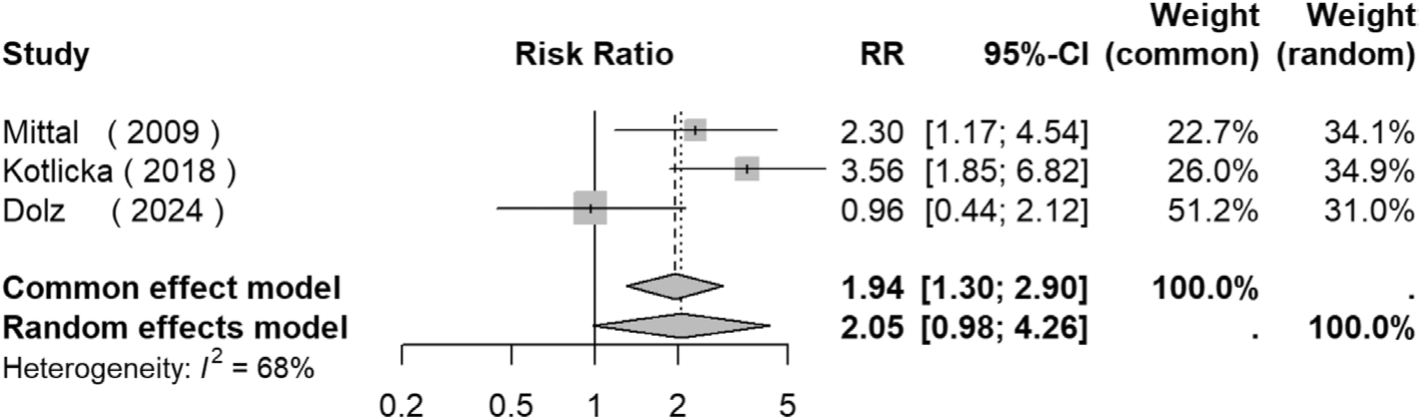
Forest plot of studies measuring the prevalence of obstetric complications between clinical high risk for psychosis subjects and first‐episode psychosis patients.

The meta‐analysis showed that no overall differences were found between the OCs prevalence in the two groups of patients: RR = 0.90 (95% CI: 0.70, 1.17, *Z* = −0.79, *p* = 0.43; Figure 4). Also, no asymmetry was found in either the funnel plots drawn for the meta‐analysis or the Egger's tests that were performed (Figure S2).

## 4 Discussion

To our knowledge, this is the first systematic review and meta‐analysis conducted regarding the prevalence of OCs in CHR for psychosis subjects and their potential role in the transition to a full psychotic disorder. Our meta‐analytic results show a higher prevalence of OCs in CHR subjects compared to controls. Moreover, the meta‐analysis showed there was a trend toward a positive association between OCs and transition to a psychotic disorder in CHR patients, although it was only possible to include three studies in this analysis.

OCs are frequent in cases of psychosis, although it is difficult to know their total prevalence. In general, around 25%–30% of births later diagnosed with schizophrenia involve at least one Lewis–Murray complication [15]. In the general population, 15% of all pregnant women will develop a potentially life‐threatening complication that calls for skilled care, and some will require a major obstetrical intervention to survive, according to the World Health Organization [55]. It is also known that advanced maternal age increases the prevalence of OCs [56], and later pregnancies are a phenomenon that has become more common in Western countries [57, 58]. Additionally, certain OCs can lead to others. For instance, first trimester vaginal bleeding may increase the risk of severe preeclampsia or preterm birth [34]. All told, OCs are a frequent event in pregnancies that can impact with different degrees of severity both the mother and the baby.

Regarding the types of OCs found in the general population, in Europe, 26% of all pregnancies include cesarian delivery [59]. Looking at OCs related to gestational age, preterm birth among live births is found in 6.9% of European pregnancies, and postterm birth in < 1% [59]. These OCs were also the most prevalent ones in our review, in which complications associated with delivery were the most frequent. Specifically, cesarian delivery ranged between 4.8% and 24.2%. Moreover, abnormal gestational age (an OC linked to fetal growth and development) was found in 7.7%–29.2% of the subjects.

In children, it is known that OCs are linked to psychotic disorders and schizophrenia [15, 17, 18] and to genetic high‐risk groups for psychosis [60] compared to controls. OCs are considered to be a risk factor with a RR of 1.45 for a CHR state compared to controls in our meta‐analysis.

Another of our findings was that, according to the meta‐analysis of three studies, exposure to OCs showed a trend toward a positive association with transition to a psychotic disorder in CHR patients. In fact, OCs have been included in an environmental risk score for psychosis in asymptomatic individuals [19] and in a polienviromic risk score of conversion to psychosis in familial high‐risk subjects [61]. Nevertheless, psychosis risk calculators in CHR subjects have not included OCs [62].

Our secondary analysis and results showed no differences between CHR and FEP/FES patients. OCs could be a risk factor for a broad spectrum of psychosis‐related conditions ranging from at‐risk state (genetic or clinical) to a full‐blown disorder. Indeed, psychotic experiences in the general population [63] are common from childhood to young adulthood and have been related to perinatal events [64]. So, the similar prevalence might underlie an environmental risk factor related to the psychosis spectrum, with other second‐hit risk factors later leading to the development of a full psychotic disorder.

Some limitations of this review need to be taken into account. First, some of the articles included small sample sizes. Second, the available studies do not allow conclusions regarding specific types of OCs and their relation to the development of psychosis. Third, some of the studies could not be included in the meta‐analysis because they offered incomplete data and we were not able to obtain some of this information even after contacting the authors. Fourth, most of the studies included the Lewis–Murray scale, which was established almost 35 years ago and focused on the most prevalent OCs at that time. Some of these have changed over time. For instance, rhesus incompatibility has become much less common in recent decades, while prematurity has increased considerably, mainly as a consequence of new and better treatments aimed at promoting the survival of the fetus. Regarding the duration of pregnancy, both prematurity and post‐term delivery are lumped together in the Lewis–Murray scale as abnormal gestational age. However, the effects of each of these involve different pathophysiological mechanisms. The same applies to cesarean section, which over the years has changed its prevalence due to external circumstances not related to fetal health [65]. Moreover, other pregnancy complications such as gestational diabetes were not specifically included in this systematic review, while other factors that could impact pregnancy, such as the existence of a psychiatric diagnosis in mothers or psychotropic treatment during pregnancy, were not taken into account in the systematic search for this review. Lastly, CHR criteria varied between the included studies, with different standards such as SIPS, CROP, and CAARMS being used.

The strengths of the article include the fact that the review followed the internationally accepted PRISMA criteria and was registered in the PROSPERO database. Additionally, the articles which were included showed homogeneity in terms of the criteria used for assessing OCs and are high quality studies according to the Newcastle–Ottawa scale.

## 5 Conclusions

To our knowledge, this is the first systematic and meta‐analytic review specifically evaluating the prevalence of OCs in CHR subjects, and we observed an increased risk in this population compared to controls. Our findings suggest that OCs may be a risk factor for a broad spectrum of conditions ranging from CHR risk to full‐blown psychotic disorders.

Our meta‐analysis showed a tendency toward a positive association between OCs and transition to psychosis, though more studies would be needed to confirm this result. Nevertheless, it could be beneficial for early detection of psychosis services to systematically assess OCs in help‐seeking individuals, and to evaluate psychosis risk in adolescents with OCs, as well as to promote preventive intervention strategies.

## Author Contributions

Inmaculada Baeza had the idea to do this review and registered the protocol in PROSPERO. Inmaculada Baeza and Jordina Tor performed the literature search. Inmaculada Baeza, Jordina Tor, and Clemente García‐Rizo wrote the draft. All the authors critically revised the work, read and approved the final manuscript.

## Conflicts of Interest

Inmaculada Baeza has received honoraria or travel support from Otsuka‐Lundbeck and Angelini and grants from the Spanish Ministry of Science, Innovation and Universities, Carlos III Health Institute, Fundación Alicia Koplowitz, and National Drugs Plan (Ministry of Health). Montserrat Dolz has participated as principal site investigator in two Janssen‐Cilag, S.A., clinical trials. She has received grants as principal investigator from the Spanish Ministry of Health, Instituto de Salud Carlos III and the Alicia Koplowitz foundation. She has received support to attend conferences or speaker fees from Shire, Janssen, and Otsuka‐Lundbeck. Gisela Sugranyes has received research grants from the Spanish Ministry of Health, Instituto de Salud Carlos III “Health Research Fund”/FEDER; the European Commission; the Brain and Behaviour Research Foundation; Department of Health, Government of Catalonia; La Marató TV3; the Alicia Koplowitz Foundation, Pons Bartran and Maria and Nuria Cunillera legacies, and INVESTIGO‐AGAUR (Generalitat de Catalunya). Clemente García‐Rizo has received grants from/or served as consultant, advisor or speaker for the following entities Adamed, Angelini, Casen‐Recordati, Janssen‐Cilag, Lunbeck and Newron. The other authors declare no conflicts of interest.

## Supporting information


Data S1.

## Data Availability

The data that support the findings of this study are available from the corresponding author upon reasonable request.
