# Supplementary material for: Impact of Obstetric Complications in Subjects at Clinical High Risk for Psychosis: A Systematic Review and Meta‐Analysis
Source: Acta Psychiatr Scand. 2025 May 4;152(2):81–93. doi: 10.1111/acps.13816 (PMC12213012; doi:10.1111/acps.13816)
Supplement: Supplementary file 1 — Data S1. [file ACPS-152-81-s001.docx]

**Impact of obstetric complications in subjects at Clinical High Risk for Psychosis: A systematic review and meta-analysis**

**Supplementary material**

Inmaculada Baeza, Jordina Tor, Elena de la Serna, Gisela Sugranyes, Fàtima Crispi, Montserrat Izquierdo Renau, Marta del Olmo, Montserrat Dolz, Clemente García-Rizo

Corresponding author: Inmaculada Baeza

ibaeza@clinic.cat

Contents

[Supplemental Table 1. Description of the Newcastle-Ottawa scale scores for the case-control studies included in the review (N=4). 2](#_Toc174522678)

[Supplemental Table 2. Description of the Newcastle-Ottawa scale scores for the cohort studies included in the review (N=6). 3](#_Toc174522679)

[Supplemental Table 3. Percentages of obstetric complications (any) among the studies reviewed. 4](#_Toc174522680)

[Supplemental Table 4. Percentages of Obstetric complications (definite) among the studies reviewed. 5](#_Toc174522681)

[Supplemental Figure 1. Funnel plot and Egger’s test of the meta-analysis including studies of obstetric complications prevalence in Clinical High Risk for psychosis participants vs. Healthy controls. 6](#_Toc174522682)

[Supplemental Figure 2. Funnel plot and Egger’s test of the meta-analysis including studies of obstetric complications prevalence in Clinical High Risk for psychosis participants vs. First episode of psychosis/schizophrenia patients. 7](#_Toc174522683)

# **Supplemental Table 1**. Description of the Newcastle-Ottawa scale scores for the case-control studies included in the review (N=4).

|  | **SELECTION** | | | | **COMPARABILITY** | **OUTCOME** | | |  |
| --- | --- | --- | --- | --- | --- | --- | --- | --- | --- |
| **Study** | **Case definition** | **Cases representativeness** | **Control selection** | **Control definition** | **Cases and controls comparability**  **(a-OCS assessment;**  **b-psychopathology scales)** | **Exposure ascertainment** | **Same ascertainment** | **Non-response rate** | **Score** |
| Ballon et al, 2008^27^ | * | * | * | * | * | 1c | * | 3b | 6 |
| Kotlicka-Antczak et al, 2014^28^ | * | * | * | * | * | 1c | * | * | 7 |
| Dolz et al, 2018^29^ | * | * | * | * | ** | 1c | * | * | 8 |
| Dolz et al, 2024^30^ | * | * | * | * | ** | 1c | * | * | 8 |

# **Supplemental Table 2.** Description of the Newcastle-Ottawa scale scores for the cohort studies included in the review (N=6).

|  | **SELECTION** | | | | **COMPARABILITY** | **OUTCOME** | | |  |
| --- | --- | --- | --- | --- | --- | --- | --- | --- | --- |
| **Study** | **Exposed cohort representativeness** | **Non exposed cohort ^a^** | **Exposure ascertainment** | **Outcome at start**  **(psychotic disorder)** | **Cohorts comparability**  **(a-OCs; b-psychopathology at follow-up)** | **Outcome assessment** | **FU duration**  **(12 months)** | **FU cohort**  **(>80%)** | **Trial score** |
| Yun et al, 2005^32^ | * | 2d | * | * | ** | * | * | * | 7 |
| Mittal et al, 2009^37^ | * | 2d | * | * | ** | * | * | * | 8 |
| Preti et al, 2012^38^ | * | 2d | * | * | ** | * | * | 3c | 7 |
| Korkeila et al, 2013^39^ | * | 2d | * | * | ** | * | _ | _ | 6 |
| Kotlicka-Antczak et al, 2018^36^ | * | 2d | * | * | ** | * | * | * | 8 |
| Dolz et al, 2024^30^ | * | 2d | * | * | ** | * | * | * | 8 |

FU: follow-up; OCS: obstetric complications

^a^An additional response to the second parameter was added (d: non-existence of the non-exposed cohort, rated 0 points) following Pozzi et al, 2022.

[Pozzi M, Ferrentino RI, Scrinzi G, Scavone C, Capuano A, Radice S et al. Weight and body mass index increase in children and adolescents exposed to antipsychotic drugs in non-interventional settings: a meta-analysis and meta-regression.](https://pubmed.ncbi.nlm.nih.gov/32617775/) *Eur Child Adolesc Psychiatry*. 2022; 31(1):21-37. doi: 10.1007/s00787-020-01582-9.

# **Supplemental Table 3**. Percentages of obstetric complications (any) among the studies reviewed.

|  | Total sample  CHR | Group A | Group B | Group C |
| --- | --- | --- | --- | --- |
| Yun et al, 2005 | 74 | 23.7 | 53.9 | 61.1 |
| Baloon et al, 2009 | 52 | 3,8 | 11.5 | 49.9 |
| Kotlicka-Antczak et al, 2014 | 66 | 9.1 | 18.2 | 45.5 |
| Kotlicka-Antczak et al, 2018 | 82 | 8,.4 | 24.4 | 40.6 |
| Dolz et al, 2019 | 67 | 13.4 | 28.4 | 76.1 |
| Dolz et al, 2024 | 91 | 12.1 | 24.2 | 69.2 |

(A): syphilis or rubella, rhesus isoimmunization/Rh incompatibility, severe preeclampsia, requiring hospitalization or induction of labor, and bleeding before delivery of threatened abortion

(B): twin delivery, preterm birth week less than 37 weeks, or long-term birth week of more than 42 weeks, weight at birth less than 2500 g, and any important physical abnormality

(C): premature rupture of membranes or prelabor rupture of membranes, duration of delivery more than 36 hours or less than 3 hours, umbilical cord prolapse, complicated cesarean delivery, abnormal fetal presentation, use of forceps, and being in an incubator for more than 4 weeks

# **Supplemental Table 4**. Percentages of Obstetric complications (definite) among the studies reviewed.

|  | Total sample  CHR | Group A | Group B | Group C |
| --- | --- | --- | --- | --- |
| Mital et al, 2009 | 47 | 6.3 | 10.6 | 36.2 |
| Kotlicka-Antczak et al, 2014 | 66 | 9.1 | 6.2 | 22.8 |
| Kotlicka-Antczak et al, 2018 | 82 | 8.5 | 12.2 | 22.0 |
| Dolz et al, 2019 | 67 | 11.9 | 14.9 | 31.4 |
| Dolz et al, 2024 | 91 | 11.0 | 13.2 | 17.6 |

(A): syphilis or rubella, rhesus isoimmunization/Rh incompatibility, severe preeclampsia, requiring hospitalization or induction of labor, and bleeding before delivery of threatened abortion

(B): twin delivery, preterm birth week less than 37 weeks, or long-term birth week of more than 42 weeks, weight at birth less than 2500 g, and any important physical abnormality

(C): premature rupture of membranes or prelabor rupture of membranes, duration of delivery more than 36 hours or less than 3 hours, umbilical cord prolapse, complicated cesarean delivery, abnormal fetal presentation, use of forceps, and being in an incubator for more than 4 weeks

# **Supplemental Figure 1.** Funnel plot and Egger’s test of the meta-analysis including studies of obstetric complications prevalence in Clinical High Risk for psychosis participants vs. Healthy controls.


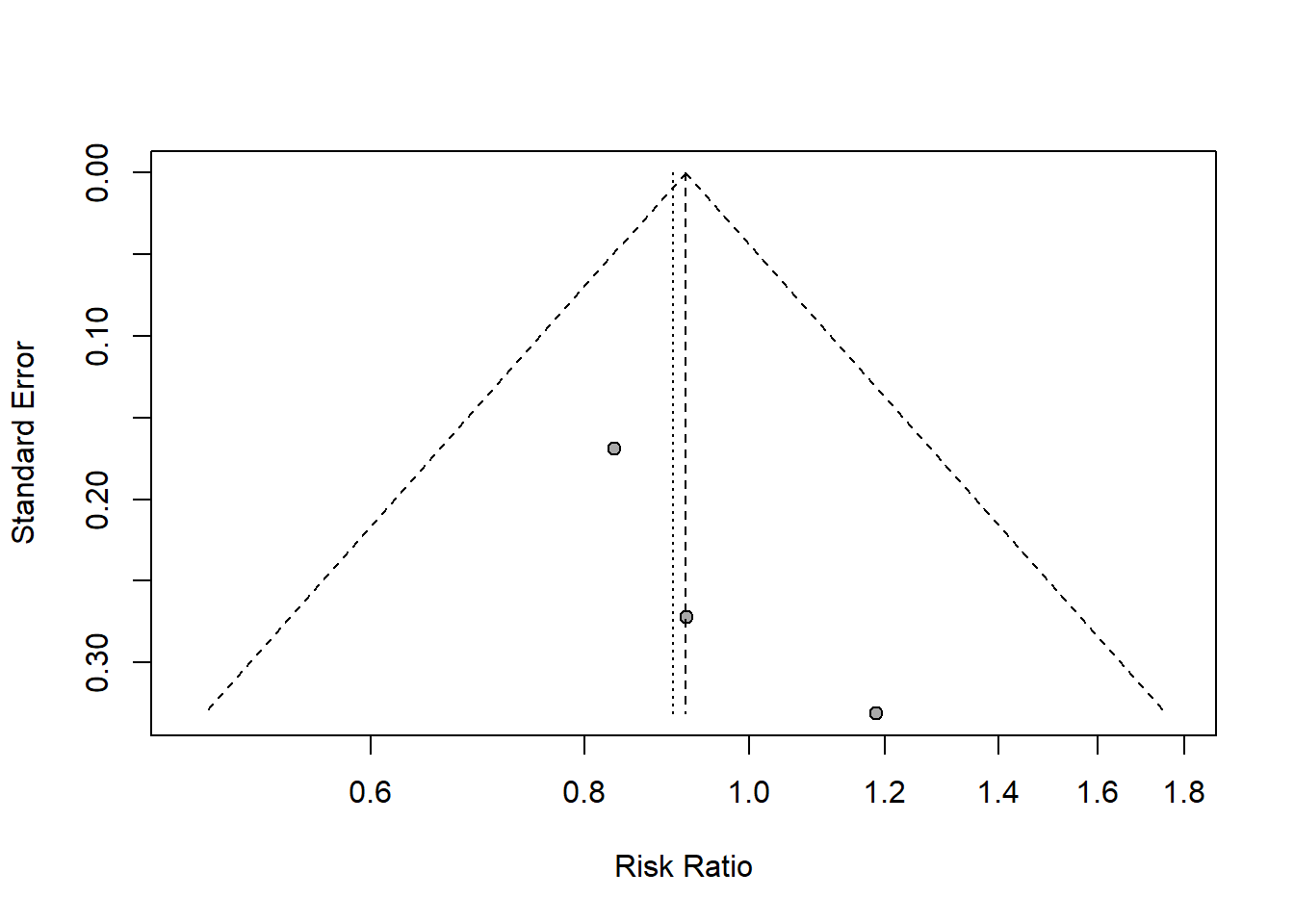


Footnote: Egger's test for funnel plot asymmetry: t = 2.67, df = 2, p-value = 0.1166

# **Supplemental Figure 2**. Funnel plot and Egger’s test of the meta-analysis including studies of obstetric complications prevalence in Clinical High Risk for psychosis participants vs. First episode of psychosis/schizophrenia patients.


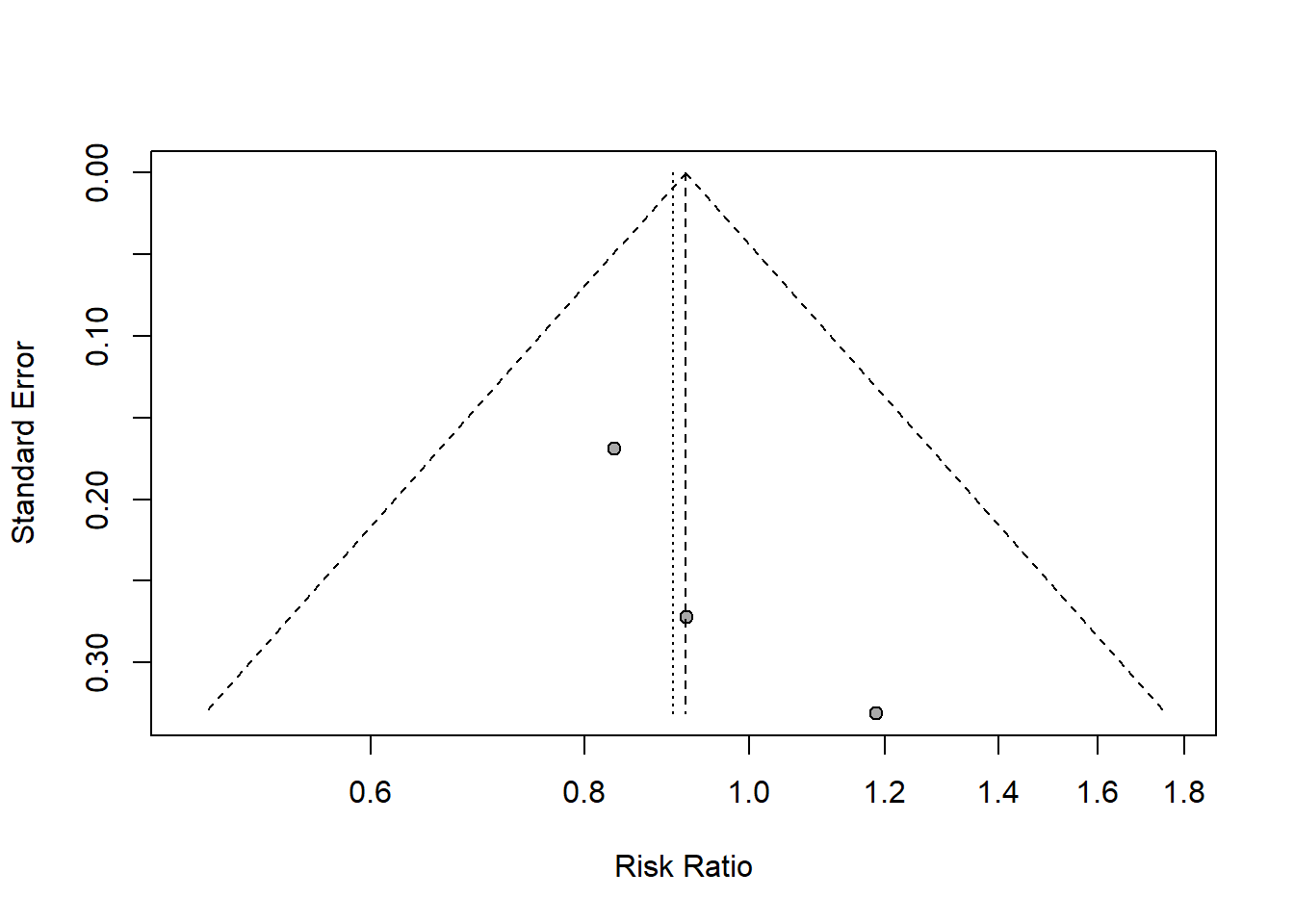


Footnote: Egger's test for funnel plot asymmetry: t = 2.44, df = 1, p-value = 0.2477.
